# Impact of Sonic Hedgehog Pathway Expression on Outcome in HPV Negative Head and Neck Carcinoma Patients after Surgery and Adjuvant Radiotherapy

**DOI:** 10.1371/journal.pone.0167665

**Published:** 2016-12-05

**Authors:** Elisabeth Enzenhofer, Thomas Parzefall, Georg Haymerle, Sven Schneider, Lorenz Kadletz, Gregor Heiduschka, Johannes Pammer, Felicitas Oberndorfer, Fritz Wrba, Benjamin Loader, Matthäus Christoph Grasl, Christos Perisanidis, Boban M. Erovic

**Affiliations:** 1 Department of Otorhinolaryngology, Head and Neck Surgery, Medical University of Vienna, Vienna, Austria; 2 Department of Clinical Pathology, Medical University of Vienna, Vienna, Austria; 3 Department of Otorhinolaryngology, Head and Neck Surgery, Rudolfstiftung, Vienna, Austria; 4 Department of Oral and Maxillofacial Surgery, Medical University of Vienna, Vienna, Austria; National Cancer Center, JAPAN

## Abstract

**Introduction:**

HPV positive patients suffering from head and neck cancer benefit from intensified radiotherapy when applied as a primary as well as an adjuvant treatment strategy. However, HPV negative patients treated with surgery and adjuvant radiotherapy lack validated prognostic biomarkers. It is therefore important to define prognostic biomarkers in this particular patient population. Especially, ´high-risk groups´ need to be defined in order to adapt treatment protocols. Since dysregulation of the sonic hedgehog pathway plays an important role in carcinogenesis, we aimed to assess whether members of the sonic hedgehog-signaling pathway may act as prognostic factors in patients with HPV negative head and neck squamous cell carcinoma.

**Materials and Methods:**

In this prospective study, pretreatment tumor biopsies of patients with head and neck squamous cell carcinoma were taken during panendoscopy (2005 to 2008). All patients were treated with surgery and postoperative radiotherapy. After assessment of HPV and p16 status, protein expression profiles of the Sonic hedgehog-signaling pathway were determined by immunohistochemistry and tissue microarray analyses in 36 HPV negative tumor biopsies. Expression profiles of Sonic hedgehog, Indian hedgehog, Patched, Smoothened, Gli-1, Gli-2 and Gli-3 were correlated with patients´ clinical data, local-control rate, disease-free as well as overall survival. Data from The Cancer Genome Atlas databank were used for external validation of our results.

**Results:**

Gli-1 (p = 0.04) and Gli-2 (p = 0.02) overexpression was significantly linked to improved overall survival of HPV negative patients. Gli-2 (p = 0.04) overexpression correlated significantly with prolonged disease-free survival. Cox-multivariate analysis showed that overexpression of Gli-2 correlated independently (HR 0.40, 95% CI 0.16–0.95, p = 0.03) with increased overall survival.

**Discussion:**

Gli-1 and Gli-2 overexpression represents a substantial prognostic factor for overall and disease-free survival in patients with locally advanced HPV negative head and neck cancer undergoing surgery and postoperative radiotherapy.

## Introduction

When combined, head and neck squamous cell carcinomas rank sixth on the list of most common human malignant tumors. Approximately two-thirds of patients present with advanced disease [1]. Radiotherapy in combination with surgery is an established treatment for advanced squamous cell carcinoma of the head and neck [2]. However, an increase in tumor cell proliferation rates after irradiation and chemotherapy contributes to tumor resistance and cell growth [3]. Thus, treatment resistance and local recurrence is frequent and pose a huge threat to overall survival. Although recent studies have shown improvement of survival in patients with advanced head and neck squamous cell carcinoma [4] after primary radiotherapy, the 5-year survival rate is less than 60% and has not changed dramatically over the last 20 years [1]. Identification of predictive as well as prognostic biomarkers are sorely needed to adapt treatment protocols and to introduce patients to effective therapies.

In clinical practice the main parameter for treatment planning and prognosis is the TNM classification [5]. However, outcomes do not always follow clinical expectations, indicating that other biomarkers than the TNM classification have to play an important role in tumor treatment and prognosis as well. In recent years, HPV positivity has been identified as a major prognostic marker in oropharyngeal squamous cell carcinoma patients. Since HPV positive patients are typically non–smokers and non–alcoholics [6] their tumors exhibit a different biologic behavior [7]. Clinical trials have shown that patients with locally advanced HPV positive head and neck cancer profit from intensified radiotherapy [8–10], not only as primary treatment, but also as adjuvant therapy. As a consequence, postoperative radiotherapy has been established as standard treatment for deemed a different patient cohort than HPV positive patients. As HPV negativity is not applicable as a prognostic biomarker, this subgroup of patients is still lacking biomarkers to adapt their treatment strategy. Thus, novel predictive markers for treatment response and clinical outcome of this particular patient cohort need to be identified [11].

In this study we firstly aimed to determine the expression pattern of members of the hedgehog-signaling pathway in HPV negative squamous cell carcinoma of the head and neck. In particular, factors involved in the hedgehog-signaling pathway control cell proliferation and differentiation during embryonic development, while they are clearly suppressed in adulthood [12,13]. It has been shown that uncontrolled upregulation of the Hedgehog signaling pathway in adults results in carcinogenesis as well as tumor survival [14]. Therefore, in this prospective, monocentric study we secondly aimed to evaluate the prognostic and predictive value of proteins of the sonic hedgehog signaling pathway in patients with locally advanced HPV negative head and neck squamous cell carcinoma undergoing surgery and adjuvant radiotherapy.

## Materials and Methods

### Patients

Tissue samples from 36 patients (7 women, 29 men, mean age 57 years, ranging from 21–76 years), with a newly diagnosed, untreated, UICC stage III and IV head and neck squamous cell carcinoma, were included. The study was prospectively planned and all tumor biopsies were obtained during diagnostic panendoscopy at the Department of Otorhinolaryngoloy, Head and Neck Surgery, Vienna, Medical University of Vienna, between the years 2005 and 2008. In this cohort all patients were treated with surgery and adjuvant radiotherapy. Follow-up was conducted every 3 months during the first two years after diagnosis. After 2 years, follow-up was conducted twice a year until a disease-free interval of 5 years was reached. Finally, patients had a once-a year routine check-up at our outpatient clinic. Demographic, clinical and pathological data were obtained from hospital records. The study was performed after obtaining approval from the institutional research ethics board (the Ethics Committee of the Medical University of Vienna and Vienna General Hospital; REB 229/2005).

### Determination of HPV and p16 Status

Immunohistochemistry as well as in situ hybridiziation was conducted on paraffin-embedded, formalin-fixed biopsies obtained during panendoscopy. Full-face formalin-fixed paraffin-embedded sections were cut in sections of 2.5μM. A validated, commercially used HPV–detection system containing genomic probes directed against the HPV genotypes 6, 11, 16, 18, 31, 33, 35, 39, 45, 51, 52, 56, 58 and 66 was used for in situ hybridization (Ventana, INFORM® Probes In Situ Hybridization (ISH) system). This kit is able to detect episomal and integrated HPV DNA and to determine high- as well as low-risk HPV-status. Data interpretation was performed with reference to the Ventana Interpretation Guide [15, 16, 17].

Staining of P16 immunohistochemistry was conducted on BenchMark Ultra platform (Ventana Medical Systems, as described elsewhere [15, 18]. Expression of nuclear p16 status was determined by three pathologists (F.O., J.P., F.W.) and scored as none, weak, moderate or strong. P16 status of the invasive tissue was counted as positive if more than 90% of the cells were scored as moderate or strong.

### Construction of tissue microarrays (TMA)

Hematoxylin and eosin (H&E) staining was performed to identify invasive tissue components. After examination of all available slides, the most representative blocks were chosen and triplicate 1.5 mm cores of the selected areas were assembled in a new paraffin TMA block.

### Immunohistochemical staining protocol

Immunohistochemical staining of sonic hedgehog proteins was performed directly after retrieval of tissue samples during panendoscopy and determination of HPV and p16 Status. In case of HPV negativity, de-waxed and rehydrated 2–3μm formalin-fixed paraffin-embedded sections were placed in Tris-EDTA buffer at pH 9.0 and heated inside a microwave oven (600W) to achieve antigen retrieval. To block unspecific binding, the samples were incubated with 5% Tris-buffered saline (TBS)/Bovine serum albumin (Sigma–Aldrich, Germany) for 1 hour at room temperature. All slides were incubated at 4°C overnight with primary antibodies directed against Smoothened (Smo), Patched (Ptch), Sonic hedgehog (Shh), Indian hedgehog (Ihh), Gli-1, Gli-3 (Santa Cruz Biotechnology, Santa Cruz, CA) and Gli-2 (Aviva Systems Biology, San Diego, CA). Negative and positive control was performed. For isotope control a secondary antibody (1:200, biotinylated-polyclonal swine anti-goat, -mouse, -rabbit; Dako, Glostrup, Denmark) was used in 1% TBS/BSA, followed by alkaline phosphatase conjugated Streptavidin-AP/TBS/BSA (1:250; Dako) each incubated for 1 hour at room temperature. Visualization was performed by fast red (Sigma, St. Louis, MO) and counterstained by hemalaun. Samples were analyzed using an Olympus BH-2 microscope (Olympus America, Center Valley, PA).

### Immunohistochemical analysis of sonic hedgehog proteins

Positive cells were quantified as a percentage of the total number of tumor cells. A cut off of 10% was taken. In particular, if >10% of all tumor cells were positive the sample was counted as positive for protein expression [19]. The mean immunoreactive score was generated from the 3 separate samples per patient. Three investigators determined the expression pattern of each marker independently (S.S., B.E., J.P.). Evaluation of protein expression was repeated at 2 different time points to avoid observer bias. Additionally, observers were blinded to patients' clinical outcomes. Staining was counted as not successful when all of the investigators concluded that tissue-staining quality was insufficient.

### Validation cohort

For external validation of our results we analyzed mRNA expression data from the RNA Seq V2 dataset in the Cancer Genome Atlas database (TCGA) [20,21]. Samples from HPV negative patients with head and neck squamous cell carcinoma were selected for analysis. Data about mRNA expression levels were available and were extracted from 2 distinct datasets within the TCGA (Head and Neck Squamous Cell carcinoma, TCGA, Provisional; Head and Neck Squamous Cell Carcinoma, TCGA, Nature 2015). In case of overlapping patients in both data sets, duplicate samples were excluded from analysis. Impact of Gli-1, Gli-2, Gli-3, Smo, Ptch1, Ihh and Shh mRNA overexpression on disease-free and overall survival was measured. Expression fold changes with a z-score of +2.0 or greater compared to the reference were considered as overexpression. HPV negativity was defined by a lack of p16 expression in ISH assay as displayed in the TCGA data set.

### Statistics

Data analysis was performed using the Statistical Package for the Social Sciences Software, (SPSS®, version 21.0; IBM Corp., Armonk, NY). Influence of nicotine abuse, history of alcohol consumption, age, sex, chemotherapy and lymph node status on expression of Gli-1 and Gli-2 was analyzed using Chi-sqare or Fisher’s exact test. Influence of tumor grading was calculated using the Kruskal-Wallis test. Overall survival (OS) was defined as the time period from primary diagnosis of head and neck cancer until death or last follow-up. Disease-free survival (DFS) was defined as the time period from patient’s first diagnosis of head and neck cancer until disease recurrence, death without recurrence or last follow-up. Local control rate was defined as the time frame from time of treatment to i) loco-regional recurrence or ii) end of observation. Curves were generated by means of the Kaplan–Meier method and group differences were tested with the log-rank statistic. Multivariate Cox proportional hazards regression analysis (backward Wald method) was used to estimate the effects of variables on survival. Hazard ratios (HR) and 95% confidence intervals (CI) were calculated. A p-value <0.05 (2-sided) was considered statistically significant.

## Results

### Patient data (Table 1)

A total of 36 patients with head and neck squamous cell carcinoma were included in the study. Demographic and pathologic data were summarized in Tables 1, 2 and 3. The most common site of primary tumor was the oropharynx (n = 13; 36.1%), followed by the hypopharynx (n = 11; 30.6%), larynx (n = 6; 16.7%) and oral cavity (n = 6; 16.7%). All patients suffered from advanced disease: Stage III: 12 (33.3%) patients and stage IV: 24 patients (66.6%). HPV high-risk status and p16 expression was determined. All tumor samples were HPV and p16 negative. The grading of 1 tissue sample was G1 (2.8%), 32 tissue samples were G2 (88.9%) and 2 tissue samples G3 (5.6%), respectively. In 1 sample the grading was not available (2.8%). 5 patients (13.9%) were non-smokers (0 pack years), 29 patients (80.5%) were smokers and of 2 patients (5.6%) no data concerning nicotine abuse was available (Tables 3 and 4). History of alcohol consumption was defined as frequent consumption of alcohol or abuse. Rate of cervical lymph node metastasis was defined as node-negative (N0) or node-positive (N+).

**Table 1:** Demographic and treatment data of 36 patients suffering from squamous cell carcinoma of the head and neck.

Characteristics	Value*
Patients	36
male	7 (19.4)
female	29 (80.6)
Age, years	
Mean	57
Median	58
Range	21–76
Tumor site	
Oral cavity	6 (16.7)
Oropharynx	13 (36.1)
Hypopharynx	11 (30.6)
Larnx	6 (16.7)
Stage	
III	12 (33.3)
IV	28 (66.6)
Disease-free survival, months	53.4
Overall survival, months	58.8
Recurrence rate	17 (47.2)
Overall mortalitiy	24 (66.6)
Disease-specific survival	
2-years	27 (75)
5-years	19 (55.9)
7-years	17 (50)
DOD	17 (47.2)
DNED	7 (19.4)
ANED	10 (27.8)
Lost to follow-up	2 (5.6)

Demographic and treatment data of 36 patients suffering of head and neck squamous cell carcinoma. Abbreviations: DOD: Died of disease; DNED: Died with not evidence of disease; ANED: alive with no evidence of disease.

*Values represent number of patients (%) except as stated otherwise.

**Table 2:** Influence of various clinicopathological factors on expression of Gli-1 in HNSCC.

Variables	Gli-1 neg.	Gli-1 pos.	p-value*
sex			
male	14 (73.7%)	14 (87.5%)	0.42
female	5 (26.3%)	2 (12.5%)	
alcohol consumption			
yes	13 (68.4%)	12 (84.6%)	0.42
no	6 (31.6%)	2 (15.4%)	
smoking			
no	3 (15.8%)	2 (14.3%)	0.91
yes	16 (84.2%)	12 (85.7%)	
age			
<60 yrs	12 (63.2%)	9 (56.3%)	0.68
≥60 yrs	7 (36.8%)	7 (43.8%)	
pN-classification			
pN0	2 (10.5%)	6 (37.5%)	0.11
pN+	17 (63.0%)	10 (62.5%)	
staging			
III	6 (31.6%)	5 (31.3%)	0.98
IV	13 (68.4%)	11 (68.8%)	
chemotherapy			
yes	3 (15.8%)	3 (18.8%)	0.82
no	16 (84.2%)	13 (81.3%)	
tumor grading			
G1	0 (0%)	1 (6.7%)	0.62
G2	18 (94.8%)	13 (86.7%)	
G3	1 (5.2%)	1 (6.7%)	

*p-value; a p-value ≤0.05 is considered as statistically significant

Influence of sex, consumption of alcohol, history of nicotine abuse, age, rate of pathological lymph nodes, staging, chemotherapy and tumor grading on expression of Gli-1. A p-value ≤ 0.05 was considered as statistically significant. None of the parameters showed a significant impact on Gli-1 expression.

**Table 3:** Influence of various clinicopathological factors on expression of Gli-2 in HNSCC.

Variables	Gli-2 neg.	Gli-2 pos.	p-value*
sex			
male	15 (75%)	14 (87.5%)	0.43
female	5 (25%)	2 (12.5%)	
alcohol consumption			
yes	15 (75%)	11 (78.6%)	0.81
no	5 (25%)	3 (21.4%)	
smoking			
no	3 (15%)	2 (14.3%)	0.95
yes	17 (85%)	12 (85.7%)	
Age			
<60 yrs	14 (70%)	8 (50%)	0.22
>60 yrs	6 (30%)	8 (50%)	
pN-classification			
pN0	2 (10.0%)	6 (37.5%)	0.11
pN+	18 (90%)	10 (62.5%)	
staging			
III	8 (40%)	4 (25%)	0.34
IV	12 (60%)	12 (75%)	
chemotherapy			
yes	3 (15%)	3 (18.8%)	0.76
no	17 (85%)	13 (81.3%)	
tumor grading			
G1	0 (0%)	1 (6.7%)	0.63
G2	19 (95%)	13 (86.7%)	
G3	1 (5%)	1 (6.7%)	

*p-value; a p-value ≤0.05 is considered as statistically significant

Association of sex, consumption of alcohol, history of nicotine abuse, age, rate of pathological lymph nodes, staging, chemotherapy and tumor grading with expression of Gli-2. None of the factors had a significant influence on Gli-2 expression. A p-value ≤ 0.05 was considered as statistically significant.

Follow-up was conducted between the years 2005 and 2015. The mean follow-up period was 8 years and 7 months (range 36.5 months– 130 months, median: 110 months). At the time of final follow–up, mean overall survival was 58.8 months (median: 55 months), whereas mean and median disease-free survival was 54 and 52 months, respectively. The 2-, 5- and 7-year disease–specific survival rates were 75%, 55.9% and 50%, respectively. The 2-, 5- and 7-year local-control rates were 63.9%, 55.9% and 50%, respectively. Overall mortality rate was 66.6% (n = 24) irrespective of the primary site of disease. The disease–specific mortality and recurrence rates were 47.2% (n = 17): Seven patients (19.4%) died without evidence of disease, 27.8% (n = 10) were alive with no evidence of disease and two patients were lost to follow-up after 36 and 58 months. At the time of last contact, both patients were alive without evidence of disease.

### Primary treatment methods

All 36 patients underwent primary surgery with curative intent and received adjuvant conventional fractionated or intensity-modulated radiation treatment with a mean of 61 Gy (range 52–70 Gy). In case of macroscopic non-in sano resection, 2 patients received postoperative intensity-modulated radiotherapy with a maximum dose of 70 Gray [22]. Eight (22.2%) patients underwent unilateral neck dissection, 26 (72.2%) patients bilateral neck dissection and 2 (5.6%) patients underwent surgery of the primary tumor without receiving a neck dissection. One patient who had no cervical lymph node metastasis chose to closely observe the cervical lymph nodes instead of surgery and the other patient denied neck dissection (Table 1). 6 (16.7%) patients received concomitant chemotherapy with 100mg/m2 cisplatin.

### Target proteins (Fig 1 and Table 4)

#### Gli-1

Overexpression of Gli-1 was present in 47.2% (n = 17) of all tissue samples. Eighteen (50%) biopsies were completely negative, whereas staining of one (2.8%) sample was counted as not successful.

**Fig 1:**
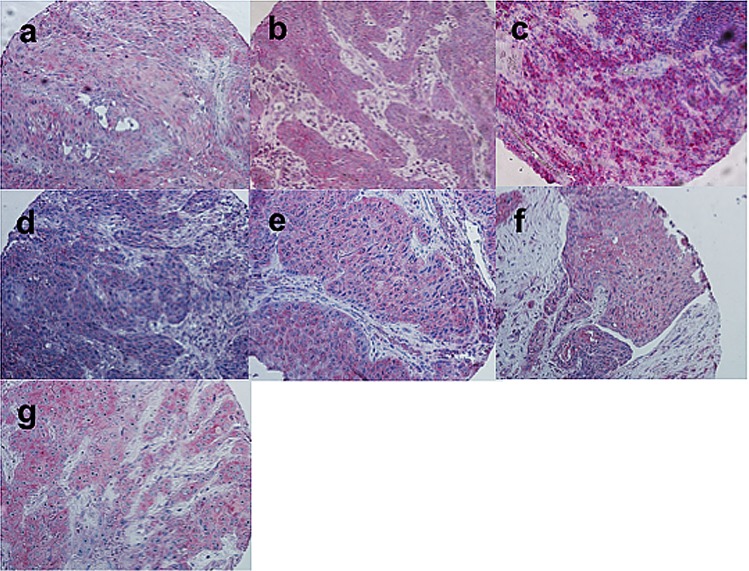
Immunostaining of a) Gli-1, b) Gli-2, c) Gli-3, d) Smoothened, e) Patched, f) Sonic hedgehog and g) Indian hedgehog in a tissue microarray of squamous cell carcinoma of the head and neck are shown. All photographs were taken at original magnification x200.

**Table 4:** Schematic representation of the tissue microarray.

Patient	Gli-1	Gli-2	Gli-3	PTCH	SHH	SMO	IHH
1	0	0	0	0	0	0	0
2	1	1	1	1	1	0	1
3	1	1	1	1	1	0	1
4	1	1	1	1	1	0	1
5	0	1	0	0	1	0	0
6	0	0	1	0	0	0	0
7	0	0	1	0	0	1	0
8	1	1	1	1	1	0	1
9	1	1	1	1	1	1	1
10	0	0	1	1	1	0	0
11	0	0	0	1	1	1	0
12	0	0	0	0	0	0	0
13	1	0	1	1	1	1	1
14	0	1	0	0	0	0	0
15	1	1	1		1		1
16	1	1	1	1	1	1	1
17	1	1	1	1	1	0	1
18	0	0	1	1	1	0	0
19	1	1	1	1	1	0	1
20	0	0	0	1	1	0	0
21	1	1	1	1	1	1	1
22	0	0	0	0	1	0	0
23	0	0	0	0	1	0	0
24	0	0	0	0	1	0	0
25	0	0	0	0	0	0	0
26	1	1	1	1	1	1	1
27	0	0	0	1	1	0	0
28	0	0	0	0	0	0	0
29	1	1	0	1	1	0	0
30		0	1	1	1	1	0
31	0	0	0	0	1	0	0
32	1	1	1	1	1	1	1
33	1	0	0	0	1	0	0
34	0	0	0	0	1	0	0
35	0	0	1	1	1	1	0
36	1	1	1	1	1	0	1

Schematic representation of the tissue microarray results. Each sample was stained for 7 different proteins of the sonic hedgehog pathway: Gli-1, Gli-2, Gli-3, Patched, Smoothened, Indian hedgehog and Sonic hedgehog. Blue squares represent negative, yellow positive and white squares loss of tissue.

#### Gli-2

In 16 (44.4%) probes Gli-2 showed a significant overexpression whereas in 20 (55.6%) biopsies staining for Gli-2 was completely absent.

#### Gli-3

Staining of Gli-3 revealed an overexpression in 55.6% (n = 20) specimens. Sixteen (44.4%) biopsies were negative.

#### Patched

Significant overexpression of Patched was observed in 58.3% (n = 21) samples. Fourteen (38.9%) biopsies were completely negative and staining was not successful in 1 (2.8%) tissue sample.

#### Smoothened

Smoothened was overexpressed in 10 (27.8%) probes, whereas 25 (69.4%) biopsies were completely negative. Staining was insufficient in 1 (2.8%) tissue sample.

#### Sonic hedgehog ligand

Staining of Shh showed weak overexpression in 33 (82.5%) specimens and seven (17.5%) samples were negative.

#### Indian hedgehog

Only 14 (38.9%) biopsies showed an overexpression of Ihh, whereas in 22 probes (61.1%) no expression of Ihh could be detected.

### Prognostic influence of protein expression

**Fig 2:**
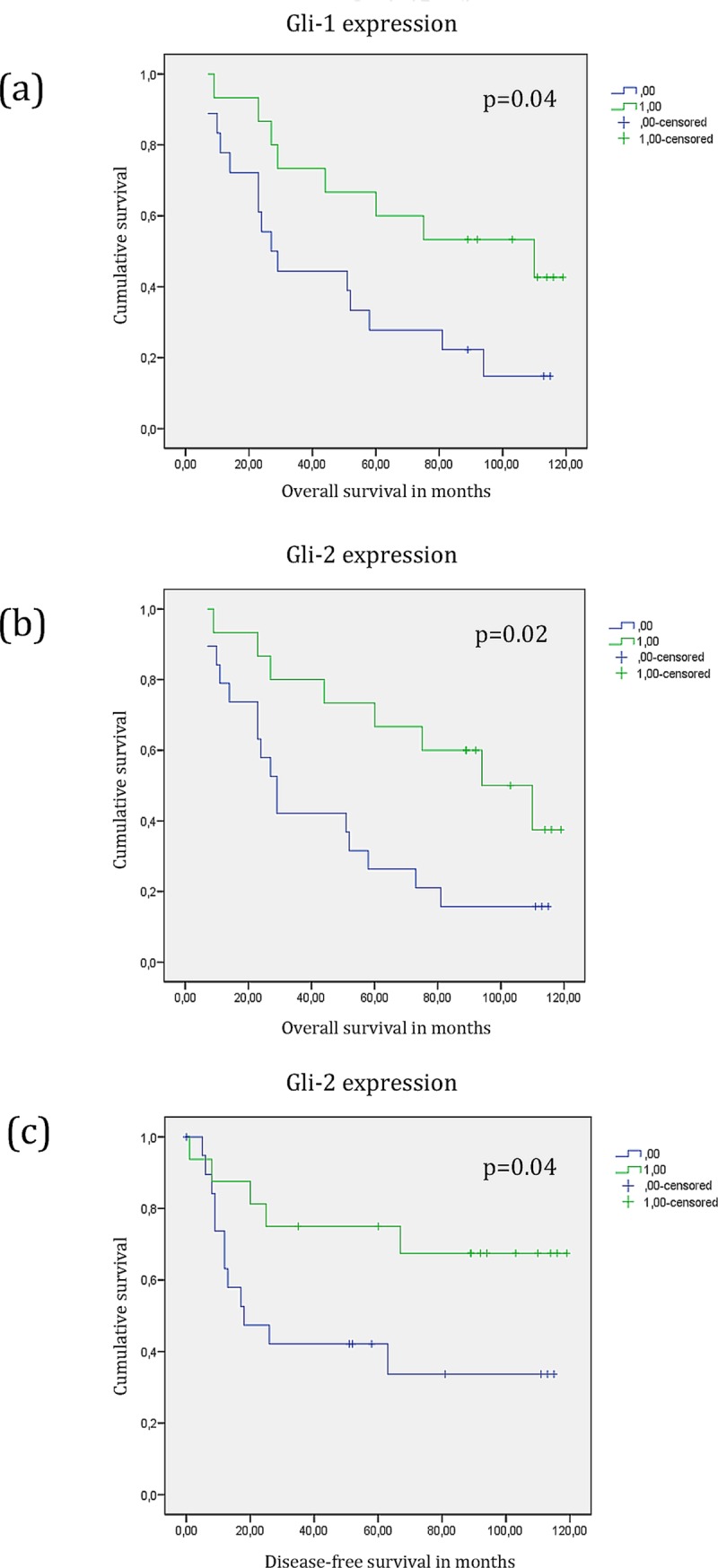
Kaplan-Meier survival curves for patients with advanced squamous cell carcinoma of the head and neck with respect to expression of Gli-1 and Gli–2. Improved overall survival correlates significantly with overexpression of Gli-1 (p = 0.04) (panel a) as well as with overexpression of Gli-2 (p = 0.02) (panel b). Overexpression of Gli-2 is significantly associated with prolonged disease-free survival (p = 0.04) (panel c).

In univariate analysis, Gli-1 overexpression was significantly associated with prolonged overall survival (p = 0.04) (Fig 2A). Kaplan-Meier univariate analysis revealed better overall survival (p = 0.02; Fig 2B) as well as better disease-free survival (p = 0.04; Fig 2C) for patients overexpressing Gli-2. All other analyzed proteins did not show any significant association with prolonged survival (OS: Gli-3: p = 0.38; Shh: p = 0.57; Smo: p = 0.60; Ihh: p = 0.25; Ptch: p = 0.26; DFS: Gli-1: p = 0.18; Gli-3: p = 0.70; Shh: p = 0.77; Smo: p = 0.85; Ihh: p = 0.44; Ptch: p = 045). None of the investigated proteins showed an impact on loco-regional control (Gli-1: p = 0.72; Gli-2: p = 0.95; Gli-3: p = 0.3; Shh: p = 0.53; Smo: p = 0.8; Ihh: p = 0.39; Ptch: p = 0.96). The impact of Gli-1, Gli-2 and TNM stage on overall survival was assessed using Cox-multivariate analysis. Overexpression of Gli-2 correlated (HR 0.40, 95% CI 0.16–0.95, p = 0.03) with increased overall survival, whereas Gli-1 expression (HR 0.64, 95% CI 0.20–2.03, p = 0.45) and TNM stage (HR 1.17, 95% CI 0.45–3.05, p = 0.74) showed no significant effect on overall survival.

Neither sex, history of nicotine or consumption of alcohol, age, tumor stage, n-status nor concomitant chemotherapy influenced expression of Gli-1 or Gli-2 (Tables 2 and 3).

### External data validation of our cohort with TCGA data

We identified 271 patients in the TCGA as HPV negative. Mean overall survival was available from all patients and was 31.7 months (range 0–211 months, median: 22 months). Data of disease-free survival was provided of 207 patients (76.4%) and mean was calculated to be 27.6 months (range 0–173 months, median: 20 months). Mean age was 61.3 years (range 19 years– 90 years, median: 62 years). 80 patients were female (29.5%) whereas 191 patients were male (70.5%). Messenger RNA expression analysis revealed that 26 patients (9.6%) showed Gli-1 upregulation, 41 patients (15.1%) had Gli-2 upregulation, 25 patients showed Gli-3 upregulation (9.2%), mRNA of Ptch1 was upregulated in 14 patients (5.2%), mRNA of Smo was upregulated in 25 patients (9.2%), mRNA of Ihh was upregulated in 5 (1.8%), whereas mRNA of Shh was upregulated in 7 patients (2.6%). Log-rank test revealed that mRNA upregulation of Gli-2 was significantly associated with prolonged overall survival (p = 0.03) as well as with disease-free survival (p = 0.04). Interestingly, upregulation of Smo mRNA was also associated with prolonged disease-free survival (p = 0.04). Upregulation of all other mRNAs did not show any significant association with either disease free survival or overall survival (data not shown).

## Discussion

Although head and neck squamous cell carcinoma is the sixth most common malignant tumor globally, overall- and disease-free survival rates did not improve substantially in recent decades. Hence, the need to deepen our knowledge about the molecular mechanisms involved in carcinogenesis is obvious to subsequently improve treatment strategies. Recently published studies identified HPV infection as a key event in the development of oropharyngeal squamous cell carcinoma and cited HPV positivity as a predictive and prognostic marker [8,9,10]. In particular, HPV positive patients demonstrate a significantly improved outcome after both primary and adjuvant radiotherapy when compared to HPV negative patients [8,9,10]. Unfortunately, patients with HPV negative head and neck cancer are still lacking established predictive and prognostic markers to *i)* predict clinical outcome and *ii)* to adapt treatment protocols and thus improve therapeutic response.

To the best of our knowledge, our prospective, monocentric cohort study is the first to show that in HPV negative head and neck cancer patients, Gli-1 and Gli-2 overexpression is significantly associated with enhanced clinical outcome after surgery and postoperative radiotherapy. In our study we examined Gli expression in a homogenous cohort of uniformly treated patients who suffered from previously untreated, locally advanced, head and neck squamous cell carcinoma. A major limitation of our study is the sample size with only 36 patients included. The low number of patients is explained by the fact that most of our patients treated at our department were HPV positive and the majority underwent primary radiotherapy. However, we validated our study by analyzing the influence of mRNA expression levels in 271 patients of the TCGA databank, which revealed a significant association of Gli-2 mRNA upregulation on prolonged overall as well as disease-free survival in this patient cohort. Interestingly, also upregulation of Smo mRNA was associated with prolonged disease-free survival. This result could not be observed in our tissue microarray analysis. This discrepancy might be due to the fact that mRNA upregulation does not necessarily correlate directly to protein expression levels since mRNA can be highly regulated and modified during posttranscriptional cellular processes.

When comparing our findings in regards to Gli protein expression to current literature, our study contradicts the results reported by Yan and colleagues [23,24]. The authors reported that Gli-2 overexpression is associated with poorer outcomes in patients with oral squamous cell carcinoma undergoing surgery with or without radiotherapy [24]. A recent study by Polanska and colleagues identified differences in protein expression in HPV negative and positive tumors [25]. In particular, HPV negative patients showed a higher expression of MMP9, MT2A, FLT1, VEGFa and POU5F compared to HPV positive patients [25]. Fertig and colleagues reported higher expression of Gli-1 in HPV negative head and neck squamous cell carcinomas. The group also compared Gli-1 mRNA upregulation in a cohort of 244 HPV negative HNSCC patients to HPV positive as well as healthy samples, which showed a higher Gli-1 mRNA status in HPV negative patients [26]. To the best of our knowledge, this is the first study highlighting the beneficial prognostic effect of Gli- proteins’ upregulation in patients with HPV negative squamous cell carcinoma of the head and neck treated with primary surgery and adjuvant radiotherapy.

When searching for an explanation for our observations, one has to consider that the functions of Gli proteins are ambiguous. Gli-1 and Gli-2 are able to act as tumor suppressors as well as tumor promoters [27,28], implying that both proteins are part of a complex network of various signaling cascades. Particularly, Gli-proteins are activated by EGF [29], K-RAS [30] or mTOR [31] in the absence of sonic hedgehog ligands and interact with the TGF-β signaling pathway in various malignant tumors [27,28]. The majority of published data claims that uncontrolled upregulation of the whole Sonic hedgehog signaling pathway results in carcinogenesis and tumor survival. However, few studies show the positive prognostic effects of cross-talks of proteins of the sonic hedgehog pathway with various growth factors. In lung adenocarcinoma, the presence of Shh and Gli-1 correlated with better overall survival, showing association with EGFR overexpression [32], whereas TGF-ß acts as a tumor promoter by transcriptionally inducing Gli-proteins [27,28] and thus mediating tumor survival. Moreover, various splicing forms of Gli-proteins were described recently, differing in structure and function from wild-type Gli proteins [33]. In particular, Gli-1 isoforms lead to tumor invasion and progression, but also play an unclear role in epithelial-mesenchymal transition [34]. Studies showed that Gli-1 acts as a promoter as well as a transcriptional repressor of E-cadherin, an epithelial marker of de-differentiation, whereas the particular functions of different isoforms of Gli-1 have not been elucidated yet [34]. Contrasting our study, Gan and colleagues described contribution to radioresistance by Gli-1 in an in-vitro tumor model [35]. It has also been shown that Gli-2 expression was associated with cell death in mice that were treated with radiation therapy for intrahepatic carcinoma [36]. Nevertheless, a recent study has shown that overexpression of survivin, which is a classical transcriptional target of Gli-2, was associated with radiosensitivity and improved survival in patients with head and neck squamous cell carcinoma [37,38]. Thus, in our cohort, the enhanced survival outcomes of patients with of Gli-overexpression may be attributed to the Gli-induced up-regulation of survivin, which subsequently might cause a positive response to postoperative radiotherapy.

In conclusion, the present study is the first to identify Gli-1 and Gli-2 as prognostic factors in patients with HPV negative head and neck cancer undergoing surgery and adjuvant radiotherapy. Our results indicate that HPV negative head and neck cancer patients with low Gli-protein expression are ´high-risk patients’ with worse overall- and disease-free survival, and subsequently should be closely monitored, so as to enable early detection of disease progression and initiate proper salvage treatment options.
